# The Importance of miRNA in the Diagnosis and Prognosis of Papillary Thyroid Cancer

**DOI:** 10.3390/jcm10204738

**Published:** 2021-10-15

**Authors:** Mariusz Rogucki, Angelika Buczyńska, Adam Jacek Krętowski, Anna Popławska-Kita

**Affiliations:** 1 Department of Endocrinology, Diabetology and Internal Medicine, Medical University of Bialystok, 15-276 Bialystok, Poland; adamkretowski@wp.pl (A.J.K.); annapoplawskakita@op.pl (A.P.-K.); 2 Clinical Research Centre, Medical University of Bialystok, 15-276 Bialystok, Poland; angelika.buczynska@umb.edu.pl

**Keywords:** miRNA, papillary thyroid cancer, diagnosis, screening

## Abstract

In recent years, the global incidence of thyroid cancer has been increasing. Despite the significant progress in the diagnostic tools applied for papillary thyroid cancer (PTC) diagnosis, commonly used methods require undergoing invasive diagnostic procedures, such as liquid biopsy, which still, in some cases, remains imprecise. In this case, novel screening and diagnostic biomarkers are still being evaluated using highly specialized techniques, which could increase PTC detection. Currently, a number of genes and proteins associated with PTC development are currently under investigation to assess their clinical utility. Accordingly, a literature search was undertaken to collect novel information about the diagnosis of and prognosis for PTC with a particular emphasis on the role of microRNA (miRNA) evaluation. The early identification of novel biomarkers is essential for facilitating appropriate therapeutic decisions. Moreover, the evaluation of plasma- and serum-derived miRNA measurements could be considered as equivalent thyroid cancer screening tools in the future. On the other hand, the PTC pathogenesis could be evaluated further with the use of miRNA evaluation, which may bring novel insights for potential medical target determination.

## 1. Introduction

In recent years, incidences of thyroid cancer have been increasing worldwide [1]. Between 1992 and 2017, the incidence of TC in the USA increased from 5.7 to 13.3 cases per 100,000 people [2]. Worldwide, nearly 300,000 cases of TC are diagnosed annually, causing nearly 40,000 deaths [3]. The estimated median age of onset is 50 years [3]. TC is much more common in women than in men [4]. Based on demographic changes in morbidity and mortality, TC is expected to replace colorectal cancer as the fourth leading cancer diagnosed in the US in 2030 [5]. 

The most common histological type of TC is papillary thyroid cancer (PTC), accounting for approximately 80–90% of all TC cases [6,7]. For the diagnosis of PTC, the analysis of medical history, physical examinations, laboratory tests (the determination of thyrotropin (TSH), thyroid hormones, thyroglobulin and calcitonin levels), ultrasound evaluation, fine-needle aspiration biopsies (FNABs), and molecular tests detecting specific gene mutations are included in the clinical management procedure [8]. Despite beneficial treatment results, 10% of PTC patients are diagnosed with relapses or distant metastases that may lead to worse clinical prognoses [9]. 

The introduction of FNAB into routine clinical management processes became the diagnostic tool of choice for the initial evaluation of solitary thyroid nodules because of its accuracy, safety, and cost effectiveness. Although patients undergoing FNAB are informed of several complication risks, ranging from the minor, such as hematoma at the injection site or pain via ecchymosis, to the major, such as clinically significant hematoma and swelling; inadvertent punctures of the trachea, carotid artery, or jugular vein occur rarely (approximately less than 2–5% per FNAB) [10]. However, several studies have reported that the side effects are inadequate to the invasiveness of this procedure. Cappelli et al. described focal carotid intramural hematoma and cancer dissemination along the needle track observed after FNAB performance [11]. On the other hand, Zhu et al. note that the frequency of acute thyroid swelling is considered a rare complication of FNAB [12]. Undoubtedly, the sensitivity, specificity, positive predictive value, and negative predictive value of FNAB are extremely high, estimated at 90–95%. Thus, consistently obtaining adequate tissue and processing the specimens to achieve accurate cytopathological interpretation requires expertise and experience [13]. Unfortunately, up to 10% of the FNABs that are performed are undiagnostic [14]. 

Therefore, there is still a need to find a cost-effective and noninvasive PTC diagnostic method characterized by high sensitivity and specificity, determined using specific novel techniques, such as genetics, and that subsequently reduce the occurrence of unnecessary invasive procedures. In this case, many proteins and genes involved in the etiopathogenesis of PTC are under investigation for their potential diagnostic uses [15]. In this review, we outline considerations of miRNA profiling with potential applications to routine frameworks of PTC diagnosis and prognosis. As it is estimated that up to 50% of PTC surgeries are unnecessary [16], the use of miRNAs may help to increase the sensitivity and specificity of FNAB, simultaneously becoming an equivalent malignant cancer screening tool measurement in the future [15]. Moreover, the currently proposed utilization of a plasma miRNA profile expression can be a valuable component of a liquid biopsy [16]. Clearly, the identification of novel PTC biomarkers remains necessary, which would increase the accuracy of both diagnostic procedures and clinical treatment decisions while introducing the assumption of personalized medicine.

## 2. PTC miRNA-Mediated Regulation of Gene Transcription

Many studies have suggested the importance of miRNA abnormalities during PTC development [17,18,19,20]. Moreover, many studies have shown differences in the deregulation of various miRNAs in thyroid cancer, depending on its type [18,19,20,21]. In PTC, the deregulation of miR-146b, miR-221, miR-222, miR-181b, and miR-21 is particularly emphasized [22,23,24,25,26]. MiR-146a and miR-146b have modulating effects on the immune system and reduce post-transcriptional gene expressions [27]. In PTC, miR-146b expressions in neoplastic tissues may be almost 30 times higher compared to non-neoplastic tissues [28]. Increased expressions of miR-146a and miR-146b have an inhibitory effect on beta retinoic acid receptor (RARß) expression, promoting the proliferation of cancer cells [28]. It has also been shown that the overexpression of miR-146b modulates the transforming growth factor β (TGF-β) pathway through the mother, and against the decapentaplegic (SMAD) transcription factor family, via member homolog 4 (SMAD4) repression, which influences the formation of thyroid tumors [29]. A study performed by Al-Abdallah et al. showed that the tissue overexpression of miR-146b reduced the expression of the major histocompatibility complex (MHC), the class I polypeptide-related sequence A (MICA), and an activating receptor (transmembrane protein) belonging to the NKG2 family of C-type lectin-like receptors (NKG2D), which is a type C lectin receptor for natural killer (NK) T cells [30]. These disturbances in mRNA synthesis may reduce the immunogenicity of PTC [30]. Moreover, increased expressions of miR-146b were previously reported among patients with the BRAF-V600E mutation [31], which suggests a correlation between the serine/threonine kinase proto-oncogene (BRAF) and miRNA expressions [22]. MiR-146b deregulation increases the risk of angioinvasion, capsular infiltration, and metastases to lymph nodes and distant organs, which result in worse survival prognoses [32]. Other studies also indicate a significant impact of miR-146b deregulation on PTC development. It was proved that a significant increase in miRNA-146b expressions in PTC resulted in worse clinical prognoses [33,34,35]. 

The deregulation of miR-221 and miR-222 has been observed to have a significant impact on carcinogenesis [36]. MiR-221 and miR-222 are highly homologous [37]. MiR-221 increases the movement and invasion of PTC cells by inhibiting the transcription of reversion-inducing cysteine-rich protein with Kazal motifs (RECK), which is a metastasis suppressor that disrupts the epithelial–mesenchymal transition [38]. MiR-221 and miR-222 affect the transformation and proliferation of thyrocytes by inhibiting p27kip1, a cell-cycle regulator [36]. High-mobility group box 1 protein (HMGB1) is a pro-inflammatory cytokine that increases miR-221 and miR-222 expression, thus promoting carcinogenesis [39]. Many other studies have confirmed that the increased expression of miR-221 and miR-222 is associated with increased tumor dimensions and a greater tendency for the cancer to infiltrate blood vessels with surrounding tissues, which simultaneously increases the probability of metastasis to lymph nodes and distant organs [19,38,40,41]. Furthermore, the study performed by Dai et al. underlined that miR-221 overexpression should be considered a PTC recurrence risk factor (hazard ratio (HR) 1.41; 95%CI 1.14–1.95, *p* = 0.007) [23]. Accordingly, these features are associated with a worse prognosis. 

Another miRNA whose expression is increased in PTC cells is miRNA-181b [42]. A study performed by Dengfeng Li et al. showed that a reduction in miR-181b expression inhibits cell division and stimulates apoptosis by upregulating lysine 63 deubiquitinase (CYLD). Moreover, the expression of miR-181b was almost 8-fold higher in cancerous tissue compared to in healthy tissue expression [43]. In addition, the overexpression of miR-181b significantly increases the risk of cancer recurrence and lymph-node metastases [44].

One of the key miRNAs implicated in the etiopathogenesis of PTC is miR-21. The expression of this miRNA was proved to be deregulated in neoplastic tissues [45]. A study conducted by Ortiz et al. showed that the overexpression of miR-21 and the aforementioned miR-141b was caused by a lack in DNA methylation, which resulted in insufficient transcription of miR-21 and miR-141b targets [46]. The study was conducted on 50 PTC and 50 tumor-free tissues, and the miRNAs were analyzed. MiR-21 overexpression may promote tumor-cell proliferation by disrupting the Von Hippel-Lindau/phosphoinositide 3-kinase/protein kinase B (VHL/PI3K/AKT) signaling pathways [26]. In addition, the inhibition of phosphatase and tensin homolog (PTEN) expressions by miR-21 promotes cancer development [47]. In a study conducted by Sondermann et al., an increased PTC recurrence rate was found to be positively correlated with decreased miR-21 expression. The authors identified miR-9 and miR-21 with as strong a predicting value as PTC recurrence [48]. In contrast, another study indicated that decreased expressions of miR-21, which is influenced by the long noncoding RNA bone marrow stromal cell antigen 2 (BST2) interferon-stimulated positive regulator (BISPR lncRNA), increased the invasiveness of PTC cells [49]. 

The following study, performed by Wang et al., showed that miR-599 increases apoptosis and decreases PTC proliferation through the downregulation of Hey2-dependant Notch signaling pathways [50]. Accordingly, Ma et al. showed that miR-199a-5p inhibits the snail family zinc finger 1 (SNAI1). Increased expressions of SNAl1 resulted in increased PTC proliferation [51] (Table 1). 

Zhang et al. suggested that miR-145 promotes apoptosis and also inhibits proliferation and migration of PTC cells. The potential medical intervention target mapped on miR-145 could result in a direct suppression of Ras-Related Protein Rab-5C (RAB5C). Ras proteins are members of a superfamily of small hydrolase enzymes that bind to the nucleotide guanosine triphosphates (GTPases) that are involved in many aspects of cell growth control, and may be a beneficial target in future medical intervention studies [52]. In turn, overexpressions of miR-643 observed during the study performed by Yin H et al. increased PTC proliferation and inhibited apoptosis. This effect was suggested due to down-regulation of the cytochrome P450 family member 11B1 [53]. Furthermore, as shown by Zhao et al., targeting insulin receptor substrate 2 and regulating the PI3K/Akt pathway is a mechanism of the function of miR-766. Its underexpression promotes PTC progression [54]. A study that was recently performed by Hu et al. has found that miR-122-5p, through dual specificity phosphatase 4 (DUSP4) inhibition, suppresses PTC oncogenesis [55] (Table 2).

Due to the rapid development of promising miRNA evaluations when using advanced technology for the comprehensive and comparative analysis of genomes, knowledge of the potentially disturbed metabolic pathways that are related to PTC development could be enhanced. Accordingly, the knowledge of disturbances of metabolic pathways involved in PTC development may lead to the discovery of novel screening and diagnostic biomarkers. Thus, the miRNA profiling could improve PTC screenings, clinical management, treatment evaluations, and individual patient prognosis assessments by introducing personalized medicine assumptions. 

## 3. The Role of miRNAs in Fine-Needle Aspiration Biopsies

FNAB is the most frequently used diagnostic method, characterized by simplicity, high specificity, a low complication rate, and low cost [56]. However, it also has disadvantages, such as non-diagnostic or abnormal results and undefined significance in describing lesions [57]. In this case, the routine analysis of specific miRNAs would increase the sensitivity and specificity of FNAB when used for PTC diagnoses [58]. 

Castagna et al. demonstrated that a PTC diagnostic miRNA panel consisting of miR-146b, miR-221, and miR-222 would increase the diagnostic utility of FNAB [58]. The study was conducted on 174 samples obtained during FNABs from 168 patients. Another study showed that miR-181b, in combination with miR-146b, might be useful in differentiating between benign thyroid lesions and PTC lesions [59]. In a study performed on 20 malignant lesion samples and 20 samples containing benign lesions, Chen et al. showed that miR-146b could be a useful PTC-screening biomarker [60]. Santos et al. created a panel consisting of 11 miRNAs, including let-7a, miR-103, miR-125a-5p, let-7b, miR145, RNU48, miR-146b, miR152, miR-155, miR200b, and miR-181, and proved its diagnostic utility for differentiating between undefined changes obtained by FNAB examination [61]. The authors named this test mir-THYpe (miRNA-based thyroid molecular classifier for precision endocrinology). In order to validate this diagnostic procedure, 58 samples from benign tissues and 39 samples from malignant tissues were used. The proposed panel was characterized by 94.6% sensitivity, 81% specificity, a 95.9% positive predictive value, and a 76.1% negative predictive value. These results suggest that the mir-THYpe test is useful for differentiating between lesions of an undefined nature, which may reduce the number of unnecessary surgeries. 

In a similar study, Mazeh et al. [62] identified a panel of miRNAs with potential diagnostic utility for differentiating between undefined lesions in FNABs. The research material consisted of 274 samples collected from 102 patients, and the miRNA expression levels were examined using Next Generation Sequencing (NGS). The Panel consisted of 19 miRNAs: miR-146b, miRNA-146, miR-222, miR-221, miR-134, miR-34a, miR-101, miR-143, miR-144, miR-615, miR-375, miR-181b, miR-194, miR-130a, miR-199a-3p, miR-30a, miR-424, miR-148a, and miR-24. Its diagnostic usefulness was proved by its 91% sensitivity and 100% specificity, and the positive and negative predictive values were estimated at 94% and 100%, respectively. The limitations of the study included the analysis of ex vivo tissues, the selective use of malignant PTC tissues, and the coexistence of other thyroid diseases among the studied patients, which may have interfered with the obtained results. 

In a subsequent study, Labourier et al. combined DNA, mRNA, and miRNA analyses into a specific PTC diagnostic panel [63]. The research was performed on 638 samples obtained during FNABs. Samples were evaluated to detect the presence of 17 genes and 10 miRNAs: miR-29b-1-5p, miR-31-5p, miR-138-1-3p, miR-139-6p, miR-146b-5p, miR-155, miR-204-5p, miR-222-3p, miR-375, and miR-551b-3p. The authors demonstrated that the effectiveness of molecular analysis was increased when genetic and miRNA tests were combined. The diagnostic usefulness of this panel was proved by its sensitivity and specificity, which were 89% and 85%, respectively. 

The cited studies indicate that miRNA evaluations have a promising role in PTC diagnoses when combined with FNAB. It is important to underline that malignant tissues could also be differentiated from benign thyroid lesions using PTC miRNA diagnostic panels. Accordingly, a specific miRNA panel would increase both the sensitivity and specificity of FNAB, decreasing the number of undiagnostic results, and relatedly, the number of unnecessary surgeries. However, these studies are still considered preliminary. Further comparison with results obtained in groups with other thyroid malignancies and thyroid comorbidities, which may have an important impact on the isolated panel of miRNAs and subsequent diagnoses, should be performed. 

## 4. PTC Screening Utility of Selected Plasma and Serum miRNAs

miRNAs can also be efficiently isolated from plasma and serum, and a specific miRNA can be investigated for potential PTC-screening utility. In a study performed by Wang et al., a panel consisting of three miRNAs isolated from plasma—miR-346, miR-34a-5p, and miR-10a-5p—was proposed as a useful tool for PTC screening [64]. The study was conducted on 30 samples obtained from PTC patients and 30 samples collected from healthy volunteers. The area under the ROC curve (AUC) of these three-miRNA panels was calculated at 0.816, which proved its great screening utility. Moreover, this study identified three miRNAs that were consistently upregulated in the exosomes obtained from PTC-patient plasma. 

Another study performed by Liang et al. proposed two combined, plasma-isolated miRNA screening panels. The first consisted of two miRNAs: miR-16-2-3p and miR-223-5p; the second consisted of six miRNAs: miR-16-2-3p, miR34c-5p, miR223 -3p, miR223-5p, miR182-5p, and miR146b-5 [65]. The study included 24 patients during the testing phase and 91 patients during validation. This study revealed that a panel consisting of miR-223-5p, miR-34c-5p, miR101-3p, and miR-16-2-3 may be particularly useful in differentiating between malignant and benign lesions. The AUC was estimated at 0.735, with 71.43% sensitivity and 73.33% specificity. 

Dai et al. analyzed the plasma of 119 PTC patients, 51 healthy subjects, and 82 patients with benign thyroid nodules. The study showed the potential PTC-screening utility of a panel consisting of miR-485-3p and miR-4433a-5p [66]. Additionally, it has been shown that the level of miR-485-3p expression could be considered as a prognostic marker, differentiating low-risk cancer from high-risk cancer. Another study performed by Li et al. confirmed the diagnostic usefulness of these measurements, demonstrating 92.8% sensitivity and 88.9% specificity [67]. The study sample was comprised of 56 patients with PTC and 95 patients with benign thyroid nodules. The control group consisted of 10 healthy volunteers, which was a notable limitation of this research.

Many authors have emphasized the potential measurement of plasma miR-222 and miR-146b levels in the PTC screening [68,69,70]. Kondrotiene et al. analyzed the plasma levels of five miRNAs—miR-221, miR-222, miR-146b, miR-21, and miR-181b—of which miR-222 had the highest screening utility. The study included 49 patients with PTC, 23 patients with benign thyroid nodules, and 57 healthy individuals. The study showed the significant overexpression of miR-221, miR-222, miR-146b, miR-21, and miR-181b [71]. 

Furthermore, the study performed by Perdas et al. suggested that the screening panel, consisting of four miRNAs, such as let-7a, let-7c, let-7d, and let-7f, whose levels were elevated in plasma, have a higher PTC screening utility [72]. Accordingly, Ricarte-Filho et al. showed that the let-7 family affects growth and differentiation of PTCs. In particular, let-7f might attenuate a neoplastic process of RET/PTC papillary thyroid oncogenesis through impairment of MAPK signaling pathway activation [73]. Table 3 shows plasma-delivered downregulated and overregulated miRNAs than may be considered for PTC screening (Table 3).

Due to the rapid development of promising miRNA evaluation methods, the clinical effectiveness of PTC screening could be improved. These measurement methods are characterized by high sensitivity, specificity, and reproducibility. Due to of differences in the types of miRNAs reported by different authors and the relatively small number of samples and difficulties in validating the tests, there is still a need for further investigations of the PTC screening utility of miRNAs. Due to the increasing number of thyroid lesions found on ultrasound, the use of miRNA as a biomarker of PTC may help to accelerate diagnosis and treatment of PTC patients. However, additional plasma/serum measurement of miRNAs would be a practical, noninvasive method for screening and for follow-up observations after thyroidectomy.

## 5. The Importance of miRNAs in the Prognosis of the Course of Papillary Thyroid Cancer

Despite a good prognosis, the frequency of PTC recurrence is estimated at 20% [74]. Many studies indicate the potential importance of miRNAs in the prognostic assessment of PTC. In the study performed by Chen-Kai Chou et al., it was shown that the overexpression of miR-146b was associated with a significant deterioration of overall survival rates. Moreover, the overexpression of miR-146b was further correlated with an increased percentage of nodal metastases and tumor invasiveness [75]. Furthermore, the polymorphism of miR-146a-3p among patients with an increased mortality rate was observed [76]. In this study, the HR of death (after adjustments for age) was 6.21 (95% CI, 1.38-27.93; *p* = 0.006).

Moreover, miR-221 and miR-222 dysregulation was observed to be more common in patients with PTC who were also diagnosed with distant metastases [77]. The study performed by Lei et al. included 78 patients diagnosed with PTC as the study group, which was subsequently divided into two subgroups: the first group consisted of 54 patients diagnosed with relapses; the second group consisted of 24 patients with no cancer recurrence. The authors identified miR-221 as a potential biomarker for PTC relapse [23]. A study performed by Pamedityde et al. on 400 PTC tissue samples obtained from paraffin blocks showed that the overexpression of five miRNAs—miR-146b, miR-222, miR-21, miR-221, and miR-181b—occurred more frequently in recurrent PTC [78]. 

Certainly, in a meta-analysis of 18 studies concerned on the role of miRNA in PTC screening, Silaghi et al. showed that miR-146b, miR-221, and miR-222 could be considered as potential screening/prognostic biomarkers of recurrent TC, and are particularly useful when referred to PTC [79]. The authors of these studies underlined the beneficial prognostic role of miRNAs in PTC screening, diagnosis and prognosis. 

## 6. Conclusions

miRNA evaluation is a promising tool in the discovery of novel diagnostic and prognostic PTC biomarkers. In recent years, genetic determination has become one of the most rapidly developing methods, characterized by increasing diagnostic utility.

The introduction of miRNAs to PTC diagnostic procedures will improve the differentiation between benign and potentially malignant lesions. Moreover, the evaluation of plasma- and serum-derived miRNAs would be particularly beneficial in PTC screening, which is, accordingly, important to the increasing number of PTC cases. On the other hand, a more detailed understanding of the pathomechanism of miRNA activity during PTC development may lead to the discovery of novel potential medical targets, which is especially necessary for the clinical management of aggressive neoplasms. One of the limitations of the application of miRNA measurements in routine and daily clinical management is the requirement for large expenditures; simultaneously, however, the prices of these procedures are constantly decreasing alongside the development of genetic medicine. The miRNAs applied in the patients’ clinical management procedures will personalize therapeutic strategies in the future.

## Figures and Tables

**Table 1:** The influence of miRNAs on PTC.

miRNA	Influence	Reference
miR-221	Overexpression is a risk factor for PTC recurrence (HR 1.41; 95%CI 1.14-.95, *p* = 0.007)	[23]
miR-222	Overexpression increases frequency of central neck metastasis and lateral neck metastasis (*p* < 0.001 and *p* < 0.001, respectively)	[41]
miR-9 and miR-21	Reduced expression of miR-9 and miR-21 increases the risk of PTC recurrence (HR = 1.48; 95% CI 1.24–1.77, *p* < 0.001; and HR = 1.52; 95% CI 1.18–1.94, *p* = 0.001; respectively).	[48]
miR-146a and miR-146b	Overexpression predicts lymph node metastasis and PTC recurrence	[34]
miR-199a-3p	Downregulation promotes the PTC proliferation	[51]

**Table 2:** Overexpressed and underexpressed miRNAs in PTC tissues.

Overexpressed miRNAs	Underexpressed miRNAs	Origin of Samples	Reference
miR-146b-5p, miR-146b-3p		Tissues	[28]
miR-146b-5p, miR-146b-3p, miR-221-3p, miR-222-5p, miR-222-3p	miR-1179, miR-486-5p, miR-204-5p, miR-7-2-3p, miR-144-5p, miR-140-3p	Tissues	[18]
	miR-9 and miR-21	Tissues	[48]
	miR-599	Tissues	[50]
	miR-199a-5p	Tissues	[51]
	miR-145	Tissues	[52]
miR-643		Tissues and serum	[53]
	miR-766	Tissues and cell lines	[54]
	miR-122-5p	Tissues	[55]

**Table 3:** Novel potential screening biomarkers determined by miRNA profiling.

Overexpressed miRNA	Underexpressed miRNA	Origin of Samples	Reference
miR-221, miR-222, miR-146b, miR-21 and miR-181b		Plasma	[71]
miR-346, miR-34a-5p, miR-10a-5p		Plasma and tissues	[73]
miR-16-2-3p, miR-223-5p	miR-34c-5p, miR-101-3p, miR-381-3p	Plasma	[65]
let-7a, let-7c, let-7d, let-7f		Plasma	[72]

## Data Availability

Not applicable.
